# Epidemiology of clubfoot in Sweden from 2016 to 2019: A national register study

**DOI:** 10.1371/journal.pone.0260336

**Published:** 2021-12-02

**Authors:** Anna-Clara Esbjörnsson, Arne Johansson, Hanneke Andriesse, Henrik Wallander

**Affiliations:** 1 Department of Clinical Sciences Lund, Lund University, Skane University Hospital, Orthopaedics, Lund, Sweden; 2 Department of Orthopaedics, Skaraborg Hospital, Skövde, Sweden; 3 Departments of Orthopedic Surgery, Gävle Hospital, Gävle, Sweden; SickKids Research Institute, CANADA

## Abstract

**Background:**

This study aimed to estimate the birth prevalence of children born with isolated or non-isolated clubfoot in Sweden using a national clubfoot register. Secondarily we aimed to describe the clubfoot population with respect to sex, laterality, severity of deformity, comorbidity and geographic location.

**Methods:**

A national register, the Swedish Pediatric Orthopedic Quality register, was used to extract data on newborn children with clubfoot. To calculate the birth prevalence of children with isolated or non-isolated clubfoot between 1^st^ of January 2016 and 31^st^ of December 2019, we used official reports of the total number of Swedish live births from the Swedish Board of Statistics. The Pirani score and predefined signs of atypical clubfoot were used to classify clubfoot severity at birth.

**Results:**

In total 612 children with clubfoot were identified. Of these, 564 were children with isolated clubfoot, generating a birth prevalence of 1.24/1000 live births (95% confidence interval 1.15–1.35). About 8% were children with non-isolated clubfoot, increasing the birth prevalence to 1.35/1000 live births (95% confidence interval 1.25–1.46). Of the children with isolated clubfoot, 74% were boys and 47% had bilateral involvement. The children with non-isolated clubfoot had more severe foot deformities at birth and a greater proportion of clubfeet with atypical signs compared with children with isolated clubfoot.

**Conclusion:**

We have established the birth prevalence of children born with isolated or non-isolated clubfoot in Sweden based on data from a national register. Moreover, we have estimated the number of children born with atypical clubfeet in instances of both isolated and non-isolated clubfoot. These numbers may serve as a baseline for expected birth prevalence when planning clubfoot treatment and when evaluating time trends of children born with clubfoot.

## Introduction

Congenital talipes equinovarus, more commonly known as clubfoot, is a common congenital orthopedic pediatric foot deformity [1]. Clubfoot is characterized by equinus of the ankle, varus of the hindfoot, as well as cavus and forefoot adduction with associated atrophy of the calf muscles [2]. The most common presentation is isolated congenital, even though clubfoot may be associated with other conditions such as myelomeningocele or arthrogryposis [3]. The cause of clubfoot is not fully known, but a multifactorial model including both genetic and environmental factors is likely [3]. About two-thirds of the children with clubfoot are boys, and 50% have bilateral involvement. For those with unilateral involvement, there is according to some studies, a slight right-sided preponderance [4]. The Ponseti method is currently considered the gold standard for treatment of clubfoot and involves weekly strict manipulation and casting. An Achilles tenotomy is performed when there is rigid equinus and all other deformities of the foot have been corrected. This is followed by brace treatment for four to five years [5].

Global birth prevalence of children born with clubfoot varies but is often reported to be between 0.4 and 2.0/1000 live births, commonly averaged to around 1/1000 live births [1,6]. Higher rates, between 3 and 6/1000 live births are, among others, reported from Malaysia, the Polynesian population and the aboriginals in Australia [7–9]. In the Scandinavian countries during 1936–1996, the rate of children with clubfoot has been reported to be between 0.7 and 1.4/1000 live births [10–14]. The highest rate being reported from a Swedish national retrospective multicenter study from 1995 to 1996 [13].

Epidemiological studies are important to improve treatment and to ensure equal care despite residential location. The information can be used to plan and evaluate treatment procedures and to compare outcomes among different centers. Epidemiological data can be used to identify factors influencing and/or causing clubfoot. In 2015, the Swedish Pediatric Orthopedic Quality register (SPOQ) was designated as a national quality register covering five common pediatric diseases, of which clubfoot is one [15]. Of all 28 pediatric orthopedic centers treating clubfoot in Sweden, 27 (96%) agreed on reporting to SPOQ. The general aim of this national prospective total cohort register is to gain generalizable knowledge, to improve outcomes and to ensure equal care for all children born with clubfoot. In addition to the number of children with clubfoot, the severity of deformity including atypical signs and other diseases related to clubfoot have been registered from the start.

### Aim

The primary aim of this study was to estimate the number of children born with isolated or non-isolated clubfoot in Sweden from 2016 to 2019 to create a baseline for future studies on birth prevalence trends. Secondarily, we aimed to describe the clubfoot population with respect to sex, laterality, severity of deformity, comorbidity and geographic location.

## Materials and methods

At birth, upon suspicion of clubfoot, the child is referred to one of the 28 pediatric orthopedic centers treating clubfoot in Sweden. Twenty-seven of these centers report to SPOQ and the coverage of the register since 2017 is 96%. Since practically all children in Sweden are born in a hospital, there are rarely any undiagnosed cases. Register data is completed by an orthopedic surgeon or a specialized physiotherapist at diagnosis using a guided checklist. To validate the number of newborn children with clubfoot registered in SPOQ, numbers were compared with those in the Swedish national patient register (PAR). Healthcare providers in Sweden are required to report ICD-10-CM codes (Q66.0) to PAR when the children are diagnosed. The averaged national completeness, thus the agreement between PAR and SPOQ, from 2016 to 2019 was 84% [15]. Every individual living in Sweden has a personal ID number (birth date [6 digits] and a unique suffix [4 digits]). The number is exclusive to an individual and is not changed during a person’s life, making comparisons between registries possible.

### Ethics statement

The study was approved by the Swedish Ethical Review Authority, Dnr: 2019–04989. The data we have used is manually registered in a specific national quality register separate from patients’ medical records. Use of data is regulated by the Swedish patient data act, in the part that applies to national quality registers. In order to be registered in a quality register, it is required that the patient is informed and given the opportunity to opt out. The information must include that the data may be used for research after approval from a research ethical board. The research ethical board decides if consent is required or not, and if data should be anonymized. In our case the decision was made that no further information or consent is required, and that data must be anonymized.

### Participants

After a clubfoot diagnosis, the child is enrolled in SPOQ by the treating hospital. Inclusion criteria in SPOQ are diagnosis of clubfoot defined as ICD-10-CM code Q66.0; born in Sweden and having a Swedish personal ID or born abroad but not started clubfoot treatment abroad and have verified contact with the Swedish health care system within six weeks of age. Exclusion criteria in SPOQ are clubfoot treatment started abroad or born abroad and not having a Swedish personal ID number at first contact with the Swedish health care system. To be registered in SPOQ, clubfoot casting treatment must start before the age of 15 months and be completed before the age of 21 months. Children with clubfoot as part of a neurological or other disease are also registered. The gold standard for treatment of children with clubfoot in Sweden is the Ponseti method [16]. In this prospective cohort study, all 612 children with a total 905 clubfeet registered in SPOQ from the 1^st^ of January 2016 to the 31^st^ of December 2019 were included.

For our study, the following variables were extracted from SPOQ: number of children with isolated congenital clubfoot (from now on referred to as isolated clubfoot); number and description of children with clubfoot as part of another disease (from now on referred to as non-isolated clubfoot); time between birth and first assessment by an orthopedic surgeon or specialized physiotherapist; gender; uni- or bilateral involvement, if unilateral, side; Pirani score before start of treatment; and description of other diseases affecting the child’s development or treatment.

The Pirani score is a disease-specific foot deformity classification system, scoring the foot deformity from 0 (no foot deformity) to 6 (maximal foot deformity) [17–19]. In SPOQ, feet scoring 1 or above are defined as clubfeet and are possible to register. Feet scoring less than 1 (0 or 0.5) are classified as positional clubfeet or other minor foot deformity and are not possible to register in SPOQ [15].

In SPOQ, children with non- isolated clubfoot as part of another disease are reported upon entry in the register in any of the following categories: arthrogryposis multiplex congenita Q74.3, spina bifida Q05.9, congenital malformation syndromes predominantly involving limbs Q87.2, neurological diseases (not specified) or other (not specified). Numbers of non-isolated clubfoot were adjusted based on updated reports at the one-year follow-up. Orthopedic centers are also encouraged to register “other diseases potentially affecting the child’s development or treatment” and to describe the condition in their own words. This is an open question, and no guidelines for reporting are provided from the register. For assessing the presence of atypical signs, SPOQ provides several illustrations to help clinicians to report this correctly. Presence of congenital atypical clubfoot is registered upon entry and is defined by two compulsory signs: distinct cavus and deep plantar creases crossing over to the lateral side, and five additional signs: short and stubby foot, extended greater toe, deep posterior crease, distinct equinus > 60°, and short calf muscles < 1/3 of the calf’s length. In this study, atypical feet were defined as isolated clubfoot if no other diagnosis related to clubfoot was registered.

### Data and statistical analyses

Statistical analyses were performed using the Statistical Package for Social Sciences, version 25 (SPSS Inc., Chicago, IL, USA) or the interactive calculator Epitools for Wilson’s score confidence intervals (CIs) [20]. To compare the annual number of newborn children with clubfoot with the total number of live births between 1^st^ of January 2016 and 31^st^ of December 2019, we used official reports concerning native data from Statistics Sweden [21]. The birth prevalence of children with clubfoot per thousand live births was calculated as the ratio between the number of children with clubfoot born between 2016 and 2019 and the number of live births during the same period. For calculating the prevalence of children born with clubfoot, Wilson’s score CIs were estimated both for isolated and non-isolated clubfoot for both genders and for each of the six official healthcare regions in Sweden, commonly used by Health Service Authorities for administrative purposes. The birth prevalence of children born with isolated clubfoot was estimated for each of the four included years separately and summarized. Only live births are registered in SPOQ; hence, abortive cases and stillborn children were not included. Norrbotten county in Sweden was not reporting children with clubfoot to the national register between 2016 and 2019; therefore, the annual numbers of live-born children from that county, in total 9784, were subtracted from the total number of live-born children in Sweden during those years.

Demographics and disease characteristics are described using median and minimum and maximum, frequency or percent. A one-sample t-test was used to evaluate differences between the proportion of boys/ girls, uni- /bilateral involvement, and in children with unilateral involvement, between the right/ left sides. Differences in Pirani score between children with isolated clubfoot and those with non-isolated clubfoot were evaluated using the Mann–Whitney U test. Differences in the proportion of feet with atypical signs between children with isolated clubfoot and those with non-isolated clubfoot were evaluated using a t-test for independent samples. To account for the effect of bilateral disease when evaluating the difference in Pirani score and proportion of feet with atypical signs between children with isolated and those with non-isolated clubfoot, we performed a sensitivity analysis. In this sensitivity analysis, we randomly included one clubfoot from each of the children with bilateral disease. The results from the two different approaches (one or both randomly selected bilateral clubfeet) did not differ. The results presented in this article include both clubfeet in bilateral cases. Differences were considered statistically significant for *P*-values < 0.05.

## Results

From the 1^st^ of January 2016 to the 31^st^ of December 2019, 564 children with isolated clubfoot were registered generating a birth prevalence of 1.24/1000 (95% CI 1.15–1.35) live births. Forty-eight children with non-isolated clubfoot were registered over the same time period generating a birth prevalence of 0.11/1000 (95% CI 0.08–0.14). In total, 612 children with isolated or non-isolated clubfoot were registered, and the birth prevalence was estimated as 1.35/1000 (95% CI 1.25–1.46). As shown in Table 1, there were slight variations between years.

**Table 1:** Newborn children with clubfoot in Sweden from 2016 to 2019.

	Children with clubfoot, n	Live births in Sweden, n	Birth prevalence per thousand live births (95% CI)
**Isolated clubfoot**			
**2016**	144	114 884	1.25 (1.06–1.48)
**2017**	121	113 003	1.07 (0.90–1.28)
**2018**	159	113 459	1.40 (1.20–1.64)
**2019**	140	112 066	1.25 (1.06–1.47)
**Total 2016–2019**	**564**	**453 412**	**1.24 (1.15–1.35)**
**Boys, isolated clubfoot, 2016–2019**	420	233 020	1.80 (1.64–1.98)
**Girls, isolated clubfoot, 2016–2019**	144	220 392	0.65 (0.56–0.77)
**Total children non-isolated clubfoot 2016–2019**	**48**	**453 412**	**0.11 (0.08–0.14)**
**Total registered children with clubfoot 2016–2019**	**612**	**453 412**	**1.35 (1.25–1.46)**

SPOQ, Swedish Pediatric Orthopedic Quality register; n, number of children; CI, confidence intervals. Years refers to birth years.

### Isolated clubfoot

Of the 564 children with isolated clubfoot, 74% were boys (mean diff. 0.24 [95% CI 0.21–0.28]) and 53% of the children had a unilateral involvement (mean diff. 0.03 [95% CI –0.01 to 0.07]), indicating no significant statistical difference between numbers of children with uni- or bilateral involvement. There was no statistically significant difference between the numbers of children with left- (47%) or right- (53%) sided unilateral involvement (mean diff. 0.03 [95% CI –0.03 to 0.09]) (Table 2).

**Table 2:** Description of children with isolated clubfoot.

		Sex		Laterality		Side in unilateral cases	
Year	Total n of children	Boys n (%)	Girls n (%)	Bilateral n (%)	Unilateral n (%)	Uni right n (%)	Uni left n (%)
2016	144	104 (72)	40 (28)	64 (44)	80 (56)	46 (58)	34 (42)
2017	121	94 (78)	27 (22)	55 (46)	66 (55)	25 (38)	41 (62)
2018	159	119 (75)	40 (25)	79 (50)	80 (50)	48 (60)	32 (40)
2019	140	103 (74)	37 (26)	66 (47)	74 (53)	40 (54)	34 (46)
**Total**	**564**	**420 (74)** ^**a**^	**144 (26)** ^**a**^	**264 (47)** ^**b**^	**300 (53)** ^**b**^	**159 (53)** ^**c**^	**141 (47)** ^**c**^

n, numbers of children.

^a^ There were significantly more boys than girls.

^b^ No statistical difference between numbers of children with uni- or bilateral involvement.

^c^ In children with unilateral clubfoot, no statistical difference between numbers of right or left involvement was observed.

Of the 564 children diagnosed with isolated clubfoot, clinicians reported other disease or condition with possible effect on development in 25 children (4.4%): prematurity (n = 9), postnatal sepsis or infection (n = 4), hip dysplasia (n = 3), congenital heart defect (n = 3), single kidney (n = 1), skin disease (n = 1), contralateral vertical talus (n = 1), contralateral metatarsus varus adducts (n = 1), abnormal pelvis (n = 1) and missing distal phalanges toes (n = 1).

### Non-isolated clubfoot

The 48 children (with a total of 77 clubfeet) with non-isolated clubfoot were reported in combination with Arthrogryposis multiplex congenita (Q74.3), n = 8; Spina bifida (Q05.9), n = 5; congenital malformation syndromes predominantly involving limbs (Q87.2), n = 18; neurological diseases (not specified), n = 7; and other (not specified), n = 10. For one child, the disease was not classified. Of the 48 children, 52% were boys and 60% had bilateral involvement.

### Severity of clubfoot deformity

**Fig 1:**
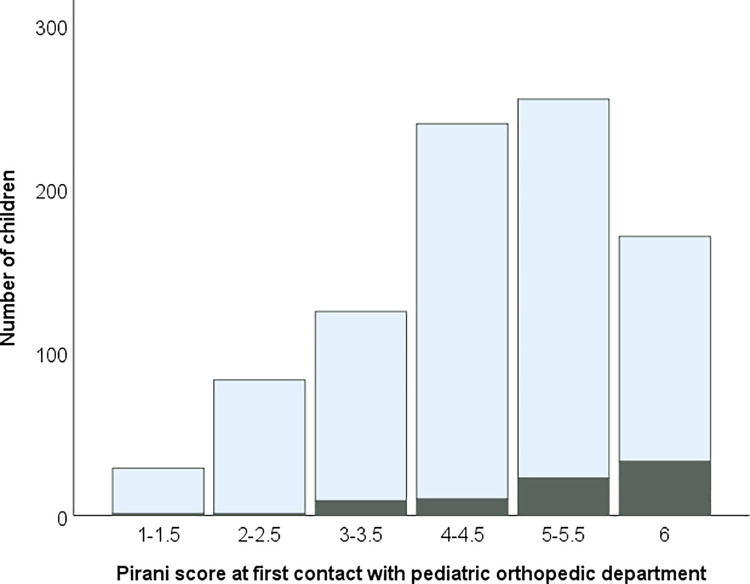
Pirani score at first contact with pediatric orthopedic department. The numbers of children with isolated clubfoot are shown in light blue, and children with clubfoot associated with other disease are shown in black.

The clubfeet (n = 828) in children with isolated clubfoot had a median Pirani score of 4.5 (range 1–6). The Pirani score in children with non-isolated clubfoot (n = 77) was significantly higher (median 5.5, range 1–6) (*P* < 0.001; Table 3, Fig 1). Of the 828 isolated clubfeet, 6% (46 feet) were classified as atypical at birth. A statistically significantly higher proportion of the 77 non-isolated clubfeet, 36% (28 feet), was classified as atypical at birth (mean diff. 0.31 (95% CI 0.25–0.37) (Table 3).

**Table 3:** Pirani score and presence of atypical signs at birth in clubfeet.

	Isolated clubfoot			Non-isolated clubfoot		
Year	Total n of clubfeet	Total score median (min, max)	Atypical n (%)	Total n clubfeet	Total score median (min, max)	Atypical n (%)
2016	208	4.5 (1, 6)	18 (9)	31	5.5 (1.5, 6)	6 (19)
2017	176	4.5 (1, 6)	13 (7)	20	5.25 (3.5, 6)	9 (45)
2018	238	4.5 (1, 6)	8 (3)	10	5.0 (3.5, 6)	3 (30)
2019	206	4.5 (1.5, 6)	7 (3)	16	6.0 (3, 6)	10 (63)
**Total**	**828**	**4.5 (1, 6)** ^**a**^	**46 (6)** ^**b**^	**77**	**5.5 (1.5, 6)** ^**a**^	**28 (36)** ^**b**^

n, number.

^a^ The Pirani score of the clubfeet in children with non-isolated clubfoot was significantly higher compared with children with isolated clubfoot.

^b^ The proportion of clubfeet with atypical signs was significantly higher in children with non-isolated clubfoot compared with children with isolated clubfoot.

#### Time to first contact with pediatric orthopedic department

**Fig 2:**
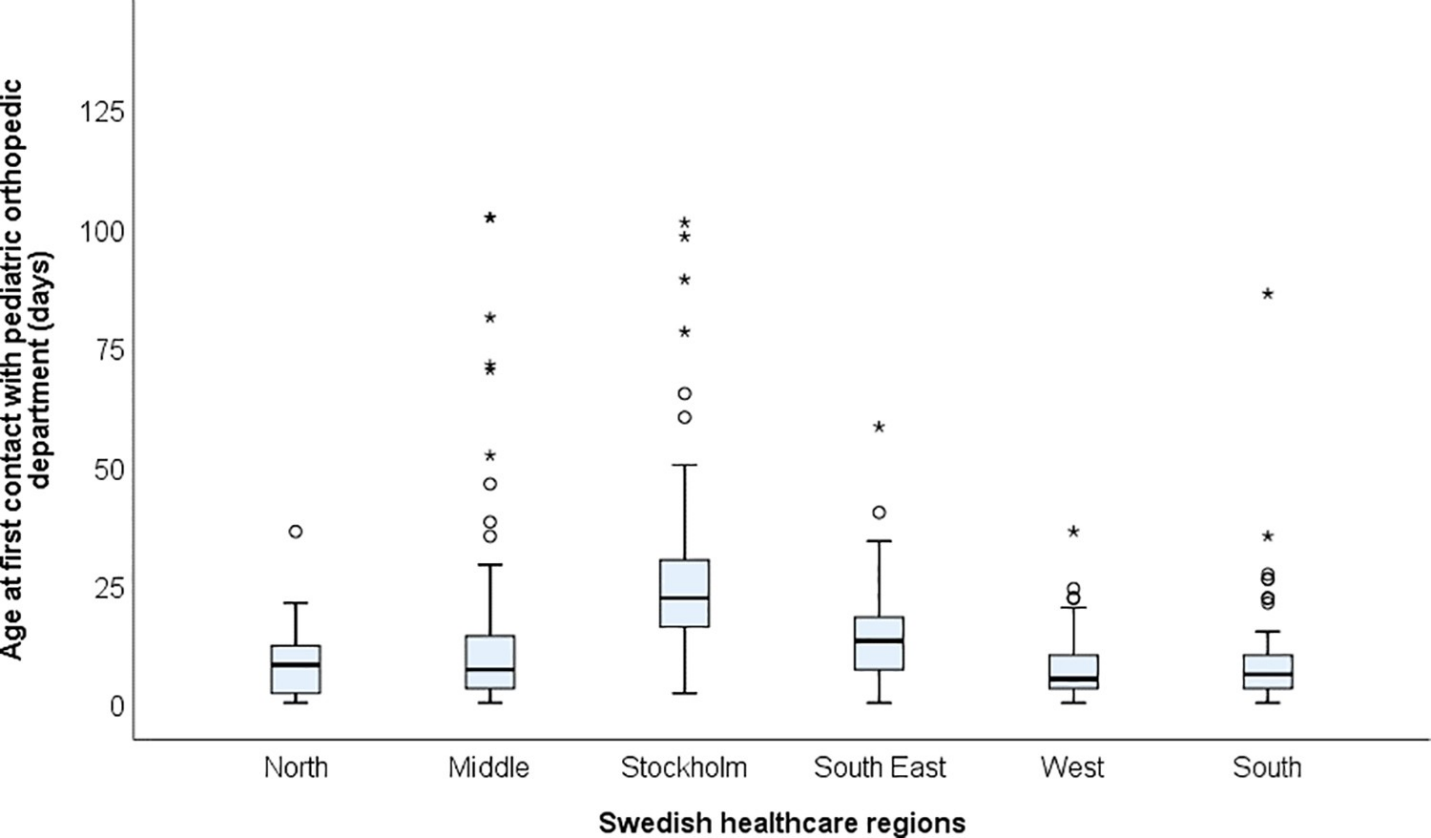
Time between birth and first contact with a pediatric orthopedic department. Children born with clubfoot from 2016 to 2019, divided between the six national health care regions. Outliers (circles) and extremes (asterisks) are also shown. One extreme at 186 days in the Middle region is not shown for scaling purposes.

The median time between birth and clubfoot diagnosis by a pediatric orthopedic surgeon was 10 days (range 0–186 days) with 67% of the children diagnosed within two weeks. There was a national variation between the six Swedish health care regions (Fig 2).

### Geographical distribution

The estimated prevalence of children with isolated or non-isolated clubfoot distributed among the six healthcare regions in Sweden is descriptively shown in Table 4. The lowest prevalence was noted in the North region and the highest in the South-East region.

**Table 4:** Children with clubfeet from 2016 to 2019 in the six healthcare regions in Sweden.

Healthcare region	Children with isolated clubfoot, n	Children with non-isolated Clubfoot, n	Total n of children with clubfoot	Live births in Sweden, n	Birth prevalence per thousand live births (95% CI)
**North**	26	3	29	27 400	1.06 (0.74–1.52)
**Middle**	120	8	128	90 742	1.41 (1.19–1.68)
**Stockholm**	122	10	132	117 444	1.12 (0.95–1.33)
**South-East**	76	10	86	46 809	1.84 (1.49–2.27)
**West**	108	10	118	86 282	1.37 (1.14–1.64)
**South**	112	7	119	84 735	1.40 (1.17–1.68)

n, number of children; CI, confidence Interval. Live births refers to all children live born in Sweden from 2016 to 2109. The birth prevalence of clubfeet is based on the total number of children with clubfeet, including both isolated and non-isolated.

## Discussion

This national Swedish register study, covering almost half a million births, found 564 children with isolated clubfoot corresponding to a birth prevalence of 1.24/1000 live births. Including the children with non-isolated clubfoot, the prevalence increased to 1.35/1000 live births. The children with non-isolated clubfoot had more severe foot deformities at birth and a greater proportion of clubfeet with atypical signs.

The estimated birth prevalence of clubfoot in children, both isolated and non-isolated, is in line with the prevalence reported by Wallander et al from 1995 to 1996 of 1.4/1000 live births [13]. Wang et al. reported a summarized birth prevalence of 1.08/1000 children with clubfoot from several countries in Europe between 1995 and 2011. In their report, our Scandinavian neighbors, Denmark (1.30/1000) and Norway (1.40/1000) reported birth prevalences of clubfoot in line with ours [1]. Comparing birth prevalence in our study with those in earlier studies covering the Swedish population from 1936 to 1990 (0.74–0.93/1000 live births), an increasing trend can be seen [10,11]. This increasing trend has also been described by Krogsgaard et al. evaluating the birth prevalence of Danish children born with clubfoot between 1978 and 1993. They showed a small increase in prevalence over time and an increased risk of being born with clubfoot in counties with higher population density [14]. However, others have described both stable and declining trends of clubfoot over time. Wang et al. reported declining trends in the proportion of children born with clubfoot in Europe between 1999 and 2011 [1], while Morris et al. described a stable trend in Europe between 2003 and 2012 [22]. Nevertheless, caution should be taken when comparing prevalences and trends of children born with clubfoot over time. Given different study protocols, lack of reporting and different inclusion and exclusion criteria, efforts should be taken to standardize these parameters [1,22–24].

Of the children with isolated clubfoot, 74% were boys, which is in line with earlier reports from Sweden [10,13] but slightly higher than in other studies from Europe reporting numbers between 65% and 72% [1,25]. Of the 48 children with non-isolated clubfoot, 52% were boys. Hence, in this group we saw an even distribution between genders. Our results are in line with other studies that also found gender differences are less pronounced in children with non-isolated clubfoot [7]. Of the children with isolated clubfoot, 47% had bilateral involvement, which harmonized with earlier reports from Sweden (46%) but was lower than reported numbers from Wang et al. (57%) [1,13]. While it is commonly described that the right side is slightly more often involved in children with unilateral clubfoot [7], this could not be statistically confirmed in our cohort. In accordance with others, we also found that bilateral involvement is more common in children with non-isolated clubfoot compared with those with isolated clubfoot [7]. Children with non-isolated clubfoot had significantly higher Pirani scores, indicating more severe deformities. We also saw a higher proportion of feet with atypical signs at birth in this group compared with children with isolated clubfoot. Syndromic clubfeet and feet with atypical signs are known to be more difficult to correct and might require more casting sessions and a slightly modified casting technique [26]. It is imperative to identify these feet early, adapt treatment and refer to a more specialized center if necessary. By including these parameters in the national register SPOQ, weaknesses in the treatment of a severely deformed clubfoot might be identified and corrected early. However, since atypical signs are registered upon diagnosis, and not after initial casting treatment, there might be missing cases. On the other hand, reporting atypical signs before start of treatment excludes those feet made complex/ atypical by improper casting.

A commonly presented rate of non-isolated clubfoot is between 10% and 13% [1,12,27]. In our cohort, about 8% of the children were diagnosed with clubfoot as part of another disease. Of the children reported as having isolated clubfoot, 4.4% had other conditions affecting development, but classified by the children’s healthcare providers as not directly associated with clubfoot. To validate the diagnosis of non-isolated clubfoot, reports upon entry were compared with reports from one-year follow-up, which classified seven additional children as having non-isolated clubfoot. This suggests to us that the 8% level of non-isolated clubfoot is low, and we expect this number to rise as the register matures. Moreover, the diagnoses that are included in non-isolated clubfoot vary between studies, a complication that is compounded by promising genetic research that may add further associated diagnoses in the future [28,29]. To make valid comparisons between studies possible, the consensus in defining isolated clubfoot as well as the subgroups of non-isolated clubfoot is essential.

Striving for an early start of clubfoot treatment after birth is generally advised even though several studies have shown positive results of Ponseti treatment in older children [30]. In our study, 67% of the children were assessed at a pediatric orthopedic center within two weeks after birth, but national variations were seen. Nowadays, clubfoot in many children is already discovered during prenatal ultrasound screening, and treatment planning can start during pregnancy. Slight variations in the birth prevalence of clubfoot were seen among the different healthcare regions with the highest prevalence reported for the South-East region. This result differs from earlier reported variations in Sweden from 1995 to 1996 where the South region had the highest prevalence [13]. The lower prevalence in the North region can be explained by low record completeness found when cases of children with clubfoot in SPOQ were compared with the national patient register for this region. In the future, when SPOQ has matured to a critical number of clubfeet, these national variations can be scrutinized statistically. In addition, the distribution of children born with non-isolated clubfoot or clubfeet with atypical signs can be further assessed.

A strength of the current study is the national prospective approach of the SPOQ register with well-predefined prerequisites for registration, excluding other subtypes of foot abnormalities or postural clubfoot. Associated diseases as well as signs of atypical clubfeet are reported upon first registration, increasing the likelihood for caregivers to address these aspects. Even so, estimated prevalence is often an underestimation of the true prevalence as there are always several missing cases. Thus, our study is not an exception. In this study only live births, and not still births or interrupted gestations, were included. Comparing numbers of children with clubfoot in SPOQ with the national patient register showed an average completeness (2016–2019) of 84%. This number is influenced by missing cases of clubfoot in SPOQ but also by wrongly diagnosed and reported cases in the national patient register. Therefore, we believe this completeness is underestimated, and further validation of the data within the register is warranted.

## Conclusion

We have established the birth prevalence of children with isolated or non-isolated clubfoot in Sweden based on data from a total population register. These numbers may serve as a baseline expected birth prevalence when planning clubfoot treatment and when evaluating time trends of children born with clubfoot.
